# Skin toxins in coral-associated *Gobiodon* species (Teleostei: Gobiidae) affect predator preference and prey survival

**DOI:** 10.1111/maec.12117

**Published:** 2014-02-01

**Authors:** Barbara Gratzer, Eva Millesi, Manfred Walzl, Juergen Herler

**Affiliations:** 1 Department of Integrative Zoology, University of ViennaVienna, Austria; 2 Department of Ethology, University of ViennaVienna, Austria

**Keywords:** Defence mechanisms, ichthyocrinotoxin, predation, predator–prey relationship, Red Sea, reef fishes

## Abstract

Predation risk is high for the many small coral reef fishes, requiring successful sheltering or other predator defence mechanisms. Coral-dwelling gobies of the genus *Gobiodon* live in close association with scleractinian corals of the genus *Acropora*. Earlier studies indicated that the low movement frequency of adult fishes and the development of skin toxins (crinotoxicity) are predation avoidance mechanisms. Although past experiments showed that predators refuse food prepared with goby skin mucus, direct predator–prey interactions have not been studied. The present study compares the toxicity levels of two crinotoxic coral gobies – *Gobiodon histrio*, representative of a conspicuously coloured species, and *Gobiodon* sp.3 with cryptic coloration – using a standard bioassay method. The results show that toxin levels of both species differ significantly shortly after mucus release but become similar over time. Predator preferences were tested experimentally in an aquarium in which the two gobies and a juvenile damselfish *Chromis viridis* were exposed to the small grouper *Epinephelus fasciatus*. Video-analysis revealed that although coral gobies are potential prey, *E. fasciatus* clearly preferred the non-toxic control fish (*C. viridis*) over *Gobiodon*. When targeting a goby, the predator did not prefer one species over the other. Contrary to our expectations that toxic gobies are generally avoided, gobies were often captured, but they were expelled quickly, repeatedly and alive. This unusual post-capture avoidance confirms that these gobies have a very good chance of surviving attacks in the field due to their skin toxins. Nonetheless, some gobies were consumed: the coral shelter may therefore also provide additional protection, with toxins protecting them mainly during movement between corals. In summary, chemical deterrence by crinotoxic fishes seems to be far more efficient in predation avoidance than in physical deterrence involving body squamation and/or strong fin spines.

## Introduction

The evolution of biological toxins, which are detrimental products to non-resistant target-organisms (Cameron 1974), and resistance to such toxins, are an evolutionary paradox (Williams *et al*. 2003). If predators do not survive encounters with toxic prey, then avoidance mechanisms cannot be learned. Similarly, there is no advantage for individual prey if it dies delivering the toxins (Williams *et al*. 2003). Nonetheless, toxicity occurs in a broad spectrum of organisms ranging from arthropods and amphibians (Daly *et al*. 2005), marine invertebrates including sponges, echinoderms and cnidarians (Halstead 1978; Bakus 1981; Cuiping *et al*. 2012), to teleost fishes (Pawlowsky 1927; Nuñez-Vazquez *et al*. 2012). This common defence strategy is used by both predators and prey, either actively by venomous animals to harm others, or passively by possessing poisonous body parts (Pawlowsky 1927). Toxins located in the skin of fishes are termed ichthyocrinotoxins (Halstead 1978). About 50 species of 13 teleost families have been reported to be crinotoxic (Cameron & Endean 1973) and some of these species are avoided by predators, suggesting a deterrent function (Cameron & Endean 1973). Ichthyocrinotoxins are common in Siluriformes (Manivasagan *et al*. 2009), Tetraodontiformes (Thomson 1964; Nuñez-Vazquez *et al*. 2012), Grammistidae (Sugiyama *et al*. 2005) and Gobiidae (Hashimoto *et al*. 1974; Lassig 1981). Although toxicity also deters parasites, fouling organisms, microbial activity, pathogens, infections (Lassig 1981; Munday *et al*. 2003), fungi, algae and settling invertebrate larvae (Cameron & Endean 1973), its effectiveness for predator avoidance – a key source of post-settlement mortality in small benthic fishes – is of particular interest. Predation and its avoidance amongst coral reef fish, including the importance of physical shelter and chemical cues, have been the subject of numerous studies (Steele 1999; Schubert *et al*. 2003; Forrester & Steele 2004; McCormick & Larson 2007; Mitchell *et al*. 2011), but studies on direct predator/prey interaction and the role of ichthyocrinotoxins are scarce.

The power of a toxin's effect on its target organism may vary with geographic region, season, dose (in relation to body weight) and the way it enters the body (Pawlowsky 1927). Crinotoxins are structurally diverse and commonly have haemolytic and sometimes also antibacterial activity (Sugiyama *et al*. 2005). This intoxicates other fishes and makes the surrounding seawater foamy (Hashimoto 1979; Shiomi *et al*. 2000). The latter effect was also reported for coral gobies of the genus *Gobiodon* Bleeker (Schubert *et al*. 2003; Dirnwoeber & Herler 2012). Such a mechanism may help to deter predators, but there are no behavioural studies on direct interactions between predator and toxic prey species, or on the reactions of both organisms. This hinders estimation of the actual survival chance of prey fishes in the field. Crinotoxins are reportedly released only during physical destruction of the stratified epithelium; no active controlling mechanism of skin toxin production and secretion has yet been found (Lassig 1981).

Coral gobies of the genus *Gobiodon* are highly specialised, showing strong associations with *Acropora* corals (Munday *et al*. 1997; Dirnwoeber & Herler 2007), with little movement between colonies (Munday 2004; Feary *et al*. 2007; Wall & Herler 2009). They defend their host corals against coral predators or competitive algae (Pratchett 2001; Dirnwoeber & Herler 2012; Dixson & Hay 2012). Habitat specialisation (Munday *et al*. 1997; Dirnwoeber & Herler 2007), association intensity with coral hosts (Herler 2007) and color – ranging from conspicuous to cryptic (Munday *et al*. 1999; Herler & Hilgers 2005) – are highly variable within the genus. Gobies are also capable of bi-directional sex-change (Munday *et al*. 1998) but show high fidelity to their breeding-partners (Wall & Herler 2009). As typical for crinotoxic fishes (Cameron & Endean 1973), they lack squamation and have sedentary (and sometimes cryptic) life habits. This combination of traits, along with small body size and poor long-distance swimming ability and speed (Lassig 1981), point to high predation pressure in the environment outside of corals (Schubert *et al*. 2003). One strategy to help reduce predation may be their toxic skin secretions (Hashimoto *et al*. 1974; Schubert *et al*. 2003). Schubert *et al*. (2003) investigated species-specific toxicity levels among six *Gobiodon* species from the Great Barrier Reef via a bioassay (Thomson 1964; Lassig 1981) and found evidence that cryptically coloured species are less toxic. Schubert *et al*. (2003) used food pellets to show that predators avoid food containing *Gobiodon*-skin extracts. Such feeding bioassay methods are usually used to investigate possible predator deterrence effects of toxic substances (Pawlik & Fenical 1992; Pisut & Pawlik 2002). However, near-natural *‘*toxicological*’* observations are sparse. Some studies used video-monitoring for a more realistic representation but dealt solely with predator–prey relationships and predation risk (Christensen 1996; Mahjoub *et al*. 2008; Staudinger *et al*. 2011). For the first time, we directly assess predator–prey interactions and study the potential benefits that fishes gain from toxicity using experimental video analysis. This approach combined continuous observation and bioassay experiments (Schubert *et al*. 2003) to identify variations in toxicity of *Gobiodon* species in the Red Sea. We also measured the persistence of toxins in the water over time to better understand predator–prey interactions, based on the statement that the toxin disappears rapidly after release (Hashimoto *et al*. 1974).

The following hypotheses were addressed: (i) the toxicity of our two sample species is lower in more cryptically coloured species, as observed by Schubert *et al*. (2003); (ii) skin toxins act as a chemical agent for predation (pre-capture) avoidance and significantly increase the encounter survival of gobies compared with that of non-toxic prey fishes; and (iii) different toxicity levels in gobies are reflected in different predation/survival rates.

## Material and Methods

### Fish collection for bioassay and predator behaviour experiments

Experiments were carried out between October and December 2010 in Dahab, Egypt (28°28′ N, 34°30′ E), Southern Gulf of Aqaba. All fishes were collected from nearby reefs. The examination of direct predator–prey-interactions was necessary because (i) goby toxins lose their effect soon after release (Hashimoto *et al*. 1974; and results below), which may even be species-specific, and (ii) it is essential to study the pre- and post-capture behaviour of predators towards toxin-releasing fishes in order to estimate the likelihood of prey survival in the field. After preliminary experiments, *Epinephelus fasciatus* was selected as a predator and *Gobiodon* spp. and *Chromis viridis* were used as prey species because all these fishes remained calm when kept in aquaria (20 l volume). *Gobiodon histrio* (Valenciennes) was selected as a vividly coloured representative (*G. his*; n = 10 for bioassay experiment; n = 18 for predation experiment), *G*. sp.3 *sensu* Herler & Hilgers (2005) as a cryptic representative (n = 9 for bioassay experiment; n = 18 for predation experiment). The small and common coral-associated damselfish *C. viridis* served as a bioassay (n = 228: 19 gobies × two individuals per trial × six time intervals) and non-toxic control species in the predation experiment (n = 18). This species is important prey for many reef-based predators (Leis & Carson-Ewart 2002). *Chromis viridis* were collected within large schools of uniformly sized individuals with similar post-settlement history. Moreover, toxin-related symptoms appeared clearly and quickly, underlining their sensitivity to *Gobiodon* skin secretions. In contrast to *Gobiodon*,*C. viridis* exhibits full-body squamation and strong fin spines for physical predation deterrence. Gobies and damselfish were collected by anaesthetisation using clove oil in a squeezing bottle (10 ml in 40 ml of 95% ethanol, diluted with 200 ml seawater), which is efficient even in low concentrations without causing permanent damage (Munday & Wilson 1997). Prey and bioassay fish size was similar for all species and experiments: mean total length (TL) in cm (±SD): bioassay experiment – *G. histrio *= 4.5 (±0.3), *G*. sp.3 = 4.5 ± (0.3), *C. viridis *= 3.7 (±0.5); predation experiment – *G. histrio *= 3.9 (±0.5) cm, *G*. sp.3 = 4.0 (±0.5), *C. viridis *= 3.9 (±0.8). Predators (n = 18; mean TL (±SD)* *= 16.3 (±2.8) cm) were caught 3 days prior to recordings using a baited handnet. Predators were starved in aerated water tanks with a continuous flow of water for approximately 3 days.

### Experimental design

#### Toxicity investigation using bioassay

One *Gobiodon* specimen per trial was gently rubbed for 20 s inside a plastic bag containing 10 ml of seawater, yielding a foamy, milky, mucous secretion (Schubert *et al*. 2003; Dirnwoeber & Herler 2012). The solution was diluted with 590 ml of seawater, mixed and put into two 300-ml buckets, into which, one *C. viridis* each (n = 38, two for each goby) was placed within 1 min after mucus yield. Using two individuals per trial instead of one (Schubert *et al*. 2003) enabled a correction of potential differences in sensitivity between bioassay specimens. Time to loss of equilibrium (Munday & Wilson 1997; Schubert *et al*. 2003) was measured using a stop-watch. After experiments, fishes were weighed, total body length measured and condition factors (Cf) calculated as Cf* *= 100*weight[g]*length^−3^ [cm]. Loss of equilibrium time of each trial was calculated as the mean loss of equilibrium time of both *C. viridis* used in each trial. *Epinephelus fasciatus* (n = 1) was also tested as a bioassay fish using the same method as described above, except for using 1800 ml of water and skin secretions of six *G. histrio* (yielding the approximately same concentration). To investigate species-specific declines of toxicity over time, the bioassay experiment was continued as described above at additional intervals (10, 30, 60, 120 and 240 min after the yield of skin mucus); the same two 300-ml mucus-seawater-solutions received per goby was used for all six intervals (1–240 min; as described above). The solution was stirred for 1 min before each trial began to ensure a homogeneous solution. If no effect on the bioassay species was apparent, the experiment was terminated after 30 min. Throughout the procedure (1–240 min) the water was not aerated because preliminary experiments showed that *C. viridis* survives >24 h in 300 ml of seawater without aeration. After experiments, fishes were returned to the reef.

#### Predator experiment using video-monitoring

The aquarium setup consisted of two containers (20-l volume each) in each of two tanks (130-l capacity each). Each camera (four in total) was fixed to a frame on top of the aquarium. A third tank provided an additional water supply and ensured consistent water quality through filtering and aeration (volume: 200 l). One predator was put into each of the four video-rigged containers and acclimatised overnight to minimise stress. During acclimatisation, aquaria were covered; covers were removed for video-recording. Video-recordings were started the next day using a Neostar (MPEG 4) digital video-recorder. One live prey specimen of each species (*G. histrio*,*G*. sp.3, *C. viridis*) was introduced to each *E. fasciatus*, whereby smaller prey specimens were paired with smaller predators and larger prey with larger predators. After each trial, all fishes were exchanged to avoid learning effects. Video observations were kept as short as possible but lasted up to 3 h depending on the motivation level of the predator. Surviving specimens were released back to the reef.

Video analysis was conducted twice to reduce variation in the assignment of behavioural categories due to varying perception in the course of surveying, using *‘*BACKUP Player*’* included in CMS (Central Management Service) Software (FLIR, Markham, Canada). Table1 lists the behavioural categories of predators and prey. Additionally, the time-span before each event was recorded, calculated as the time from prey specimens entering the water until a predator's response.

**Table 1:** Categories used for quantification of predator–prey interactions. Categories in *italics* were accompanied by strike capture

Category	Abbr.	Characterisation	Reference
Approach	A	Definite active movement of a predator in the direction of at least one prey specimen; sometimes followed by strike capture	Mahjoub *et al*. (2008)
Strike capture	SC	Sudden jump of the predator towards the prey to engulf it successfully; possibly followed by ingestion/consumption (whole prey); sometimes without preceding approach	Mahjoub *et al*. (2008)
*Consumption*	C	Additionally recorded if prey was finally ingested	Christensen (1996) Mahjoub *et al*. (2008) Staudinger *et al*. (2011)
*Manipulation time*	MT	Handling time of a prey in the predator's mouth	
*Total regurgitation*	TR	Expulsion of a prey's total body (alive or dead)	
*Rejected*	RE	Prey specimens captured but not consumed finally	

### Statistical analysis

Non-parametric statistics were applied when datasets contained one or more series of values that were not normally distributed (even after data transformation). For the bioassay experiment, potential differences in body size and condition factors of *C. viridis* test fishes and goby species-specific loss of equilibrium times of *C. viridis* were examined using Mann–Whitney *U*-tests. Toxicity decrease was explored by a one-way ANCOVA (analysis of covariance), which tests the equality of means for several univariate groups (loss of equilibrium times for all intervals). Regression analyses were used to explore potential relationships between goby toxicity and body size.

For the predator preference experiments, regression analyses were used to explore relationships between condition factor and prey size. Chi-square tests were used to directly compare frequencies of first approaches, strike captures and consumptions of *C. viridis* and both gobies. Kruskal–Wallis and Mann–Whitney *U*-tests were used to test for differences in time spans before predators approached, captured or consumed different prey species (180 min were noted in experiments in which no event occurred). A Wilcoxon test was used to compare regurgitation rates across all three prey species. Differences in the manipulation time by predators before consumption or rejection of prey fishes were tested with Mann–Whitney *U*-tests. All analyses were carried out with PAST, v. 2.16 (Hammer *et al*. 2001). To estimate survival probabilities, a survival analysis was conducted. This models the time to an event (usually death) and helps estimate the effects of certain variables (covariates) on the event. During data analysis, attention must be paid to censored data, *i.e*. data in which the event to be modelled did not occur. The Kaplan-Meier estimator and Cox-regression helped deal with these type of data. The Kaplan–Meier estimator is a non-parametric method to calculate the survival function for a specific group of observations and was used here to graphically represent the survival rates. Cox-regression, or proportional hazard model (StatSoft, Inc. 2013), commonly employed in medical studies, was used to estimate the influence of certain covariates on survival times (Staudinger & Juanes 2010). Within the predator preferences experiment, the covariates were regurgitation, manipulation time, type of species and number of species alive, as well as the condition factor and weight of predators and prey fishes. Covariates were previously checked for intercorrelation. The model may be written as:


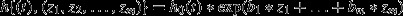


where *h*(*t,…*) is the resultant hazard (=risk of an individual to be eaten), given the values of the *m* covariates for the respective case (*z*_1_, *z*_2_,…, *z*_*m*_) and the respective survival time (*t*). The term *h*_0_(t) indicates the baseline hazard.

Both the Kaplan–Meier estimator and Cox-regression were carried out in (IBM, Armonk, NY, USA) SPSS Statistics 20 for Windows 7.

## Results

### Toxicity experiment using bioassay fishes

**Figure 1:**
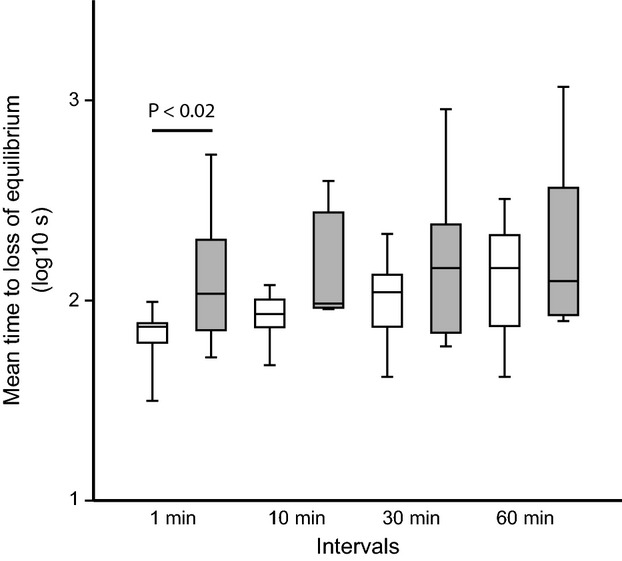
Mean time to loss of equilibrium (log10 of seconds) of *Chromis viridis* when exposed to skin toxins of *Gobiodon histrio* (white) and *Gobiodon* sp.3 (grey). Box plots represent medians (horizontal lines), upper and lower quartiles (boxes), and ranges (whiskers). The horizontal line indicates the only significant difference revealed by pair-wise Mann–Whitney *U*-tests of all intervals.

*Chromis viridis* bioassay specimens did not differ significantly between the two goby test series, neither in their weight (mean ± SD for *G. histrio* trials* *= 1.03 ± 0.4 g and for *Gobiodon* sp.3 trials* *= 1.09 ± 0.4 g; Mann–Whitney *U*-test: *P *=* *0.38) nor in their condition factor (mean ± SD* *= 1.87 ± 0.3 for *G. histrio* trials and 1.90 ± 0.3 for *G*. sp.3 trials; Mann–Whitney *U*-test: *P *=* *0.06). During all 38 trials, *C. viridis* lost its equilibrium in response to skin secretions of both gobiids. All *C. viridis* lost equilibrium within test intervals of up to 30 min; in the 60-min interval, only one individual did not lose equilibrium. In contrast, at longer intervals (120 and 240 min) a greater number of *C. viridis* did not react within the 30-min observation time (at 120 min *G. histrio*: 15%, *G*. sp.3: 11% and at 240 min *G. histrio*: 70%, *G*. sp.3: 50% of 38 *C. viridis* per interval). Therefore only data of intervals up to 60 min were used for the ANCOVA. The time to loss of equilibrium after goby mucus exposure (1-min interval) was significantly shorter for *G. histrio* than for *G*. sp.3 (median; lower and upper quartiles in seconds; *G. histrio*: 75; 56, 82; *G*. sp.3: 105; 80, 132; Mann–Whitney *U*-test for log data: U = 17, *P *=* *0.02); it was close to significance at the 10-min interval (Mann–Whitney *U*-test for log data: U = 21, *P *=* *0.056) but not significant in the longer intervals (Mann–Whitney *U*-test for log data: U > 34, *P *>* *0.4; Fig.1). ANCOVA also revealed a significantly higher basic toxicity of *G. histrio* (group mean difference: *P *=* *0.002). Toxicity clearly decreased over time and occurred equally fast in both species (homogeneity of slopes: F = 0.26, *P *=* *0.61; Fig.1). In *G. histrio*, toxicity was related to fish size: there was a significantly positive correlation between loss of equilibrium time of *C. viridis* and the length of the 10 *G. histrio* toxin donators in the most relevant test intervals (r²* *>* *0.29, P < 0.05 for the intervals 1–60 min). No such correlation was found for *G*. sp.3 in any time interval. *Epinephelus fasciatus* (n = 1) lost equilibrium after 40 min of toxin exposure.

### Predation experiment

**Figure 2:**
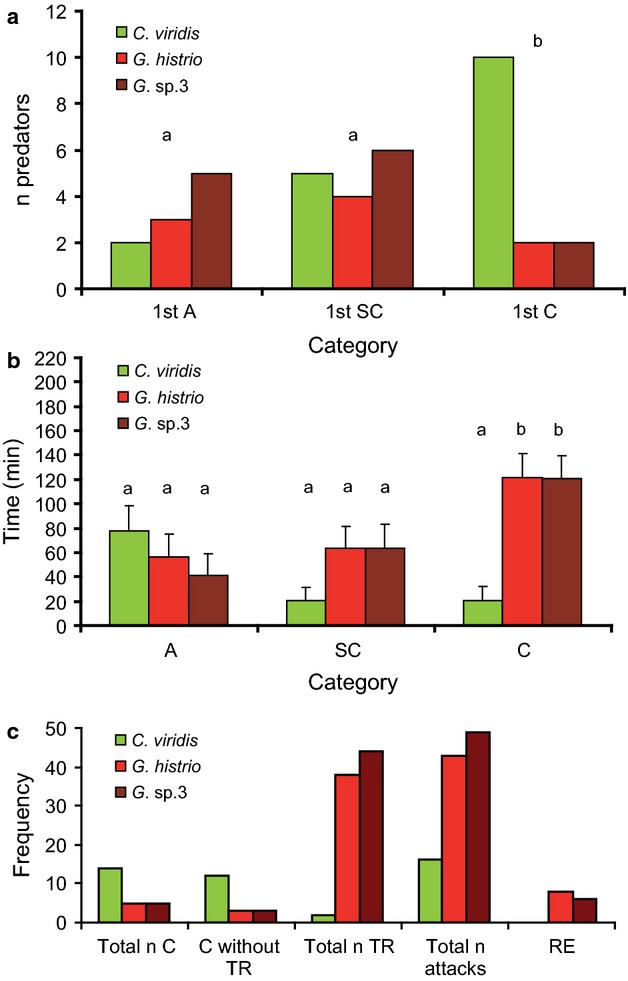
Approaches (A), strike captures (SC), consumption (C), total regurgitation (TR) and rejection (RE) of prey species. (a) Cumulative numbers of first approach, first strike capture and first consumption towards prey species of each predator (n = 15). Significant differences between categories (χ²-test of frequencies in relative percent; P < 0.001) are indicated by different letters. (b) Time span until each prey is first approached, captured or consumed by a predator. Pairwise Mann–Whitney *U*-tests were calculated only within the three categories. Significant differences (P < 0.05) are indicated with different letters. Values are means and standard errors. (c) Number of consumptions of, regurgitations of and attacks on prey individuals during all trials by 15 predators. Total *n* attacks: number of strike captures towards each prey. Note that the number of attacks on gobies is enhanced by repetitive regurgitations. RE, number of prey specimens that were captured but not ultimately consumed.

**Figure 3:**
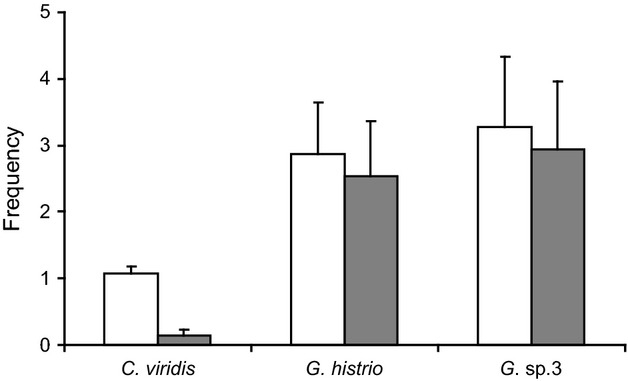
Frequencies of strike captures (white) and total regurgitations (grey) for all three prey species. Values are means and standard errors.

Condition factor and body length were negatively correlated in gobies (*G. histrio*: r^2^* *= 0.86, P < 0.001; *G*. sp.3: r^2^* *= 0.28, P < 0.05) but did not correlate in *C. viridis* (*r*^2^* *= 0.001, P* *=* *0.89). During approximately 54 h of video, 15 of 18 predators captured at least one prey fish (the three predators that showed no intention of capturing any fish were excluded from further analysis), and 14 predators consumed at least one prey fish. This yielded a total of 24 consumed fish specimens. *Chromis viridis* was consumed by 14 predators and *G. histrio* and *G*. sp.3 by five predators each. A total of 666 events were recorded. Prey preference clearly differed between pre-capture events [=approaches (A) and strike captures (SC)] and post-capture events [=either consumption (C) or regurgitation (TR) of prey]. Although prey preferences (Fig.2a) of predators during the first approach and first capture did not differ across all prey fishes, a clear preference for *C. viridis* was shown in the first consumption (A *versus* SC: χ^2^* *= 4.6, P* *=* *0.29; A *versus* C: χ^2^* *= 54.3, P < 0.001; SC *versus* C: χ^2^* *= 29.8, P < 0.001; Bonferroni-corrected). The time span (Fig.2b) before approaches and captures by predators did not differ significantly between *C. viridis* and the two gobies (pair-wise Bonferroni-corrected Mann–Whitney *U*-tests: *P *>* *0.6), whereas the time span until consumption was significantly longer in gobies than in *C. viridis* (pair-wise Bonferroni-corrected Mann–Whitney *U*-tests: P < 0.04). This confirmed that gobies, if at all, were consumed after a longer time span than *C. viridis,* additionally reflecting the preference for *C. viridis*. Twelve predators regurgitated after strike captures (Fig.3). Variation in regurgitation frequencies were species-specific and revealed a sharp contrast between *C. viridis* (more preferred) and *G. histrio* (Wilcoxon-test: P < 0.03; Bonferroni-corrected). However, the regurgitation frequency between *C. viridis* and *G*. sp.3 did not differ significantly after Bonferroni-correction of P-values (yielding a P = 0.07). The two gobies did not differ from each other (P = 1; Bonferroni-corrected).

The distribution of immediate (without regurgitation) and final consumption across the three prey species (Fig.2c) did not differ significantly (χ²* *= 1.9, d.f.* *= 3, P = 0.58; calculated from percentages of cases of 15 predators). Nonetheless, the distribution of attacks *versus* final consumption was significantly different (χ²* *= 9.9, d.f. * *= 3, P < 0.05; number of attacks normalised with respect to final consumptions). This reflects the much higher number of attacks (and regurgitations) towards gobies in comparison with ultimately consumed specimens. All captured *C. viridis* were finally consumed, regardless of regurgitation. Not consumed but initially captured prey (referred to as rejected) occurred only among *G. histrio* and *G*. sp.3 (Table2).

**Table 2:** Manipulation times (in seconds) of all three prey species from 15 predator preference experiments

Species	MT at first SC	MT per SC	Cum. MT of prey consumed	Cum. MT of prey rejected
*Chromis viridis*	25 (6, 28)	25 (6, 68)	25 (6, 68)	–
*Gobiodon histrio*	8 (5, 30)	3 (2, 12)	96 (89, 142)	11 (7, 43)
*Gobiodon* sp.3	4 (1, 45)	4 (1, 9)	96 (85, 131)	12 (8, 40)

MT = manipulation time (in a predator's mouth) in seconds; SC = strike capture; cum. = cumulative.

Values are medians with the 25% and 75% quartiles in parentheses.

Manipulation times (prey manipulation inside a predator's mouth) differed between gobies and *C. viridis* (Table2). The manipulation time for *C. viridis* following the first strike capture, the manipulation time per strike capture, and the cumulative manipulation time (sums of all manipulation times until consumption of each prey per predator) was the same because most specimens were immediately consumed. For both gobies the manipulation time following the first strike capture and the manipulation time per strike capture were similar to each other but lower compared with *C. viridis*. The cumulative manipulation times for both gobies were much longer than for *C. viridis*. The median manipulation time of gobies that were rejected (not eaten ultimately) was somewhat higher than the median manipulation time during the first strike or per strike of all specimens, but significantly lower than the cumulative manipulation time of finally consumed gobies (Mann–Whitney *U*-test: U = 18.5, P < 0.01, both species combined). The cumulative median manipulation time of consumed gobies was also significantly higher than that of consumed *C. viridis* (U = 32, P < 0.05, data of both goby species combined).

**Figure 4:**
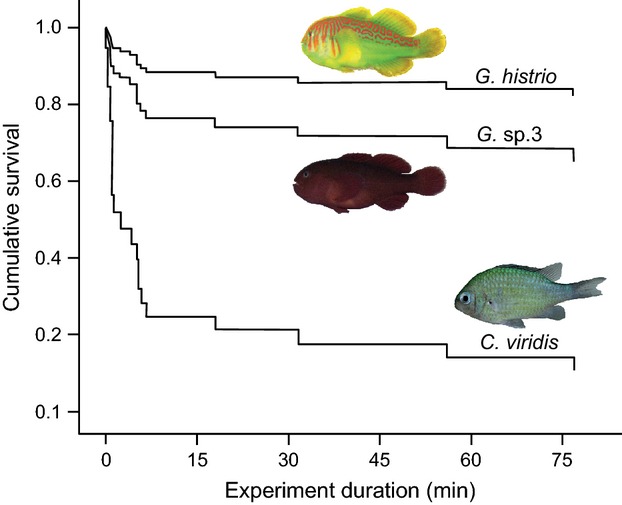
Kaplan–Mayer estimator describing the cumulative survival rates of three prey fish species exposed to the predatory grouper *Epinephelus fasciatus*: y = 1/14 (n = 14 for all predators that consumed prey).

The calculated survival rates using the Kaplan–Meier estimator showed that the cumulative survival rate for *C. viridis* was much lower than in both *Gobiodon* species, but almost equal for the two gobies (Fig.4). The Cox-regression (Table3), which was used to estimate the influence of certain covariates on survival times, revealed that *C. viridis* had a 36 times higher chance [refers to exp(B) in Table3] of being eaten as compared with *G. his-trio* and 12 times higher as compared with *G*. sp.3. The first regression model (including all specimens) also showed that the condition factor had a negative effect on survival chance, but this was mainly due to a very high condition factor of three *G. histrio* specimens. In a second regression analysis in which these specimens were excluded, the condition factor no longer had a significant effect on survival. Nevertheless, *C. viridis* still had a highly increased risk of being eaten: 11 times higher than that of *G. histrio* and five times higher than that of *G*. sp.3.

**Table 3:** Cox-regression for prey species (influence of covariates on survival). Regression analysis 1 includes all specimens, analysis 2 was calculated without three *Gobiodon histrio* specimens with unusually high condition factors (Cf). A negative value in B and a value below 1 in exp(B) indicates that the covariate reduces the likelihood of being eaten

Term	B	d.f.	Significance	exp(B)
Analysis 1				
Cf of prey	1.40	2	0.007	4.058
*Gobiodon histrio*	–3.59	1	0.001	0.028
*Gobiodon* sp.3	–2.46	1	<0.001	0.085
Analysis 2				
*Gobiodon histrio*	–2.39	1	0.002	0.092
*Gobiodon* sp.3	–1.63	1	0.003	0.197

## Discussion

Both cryptically (*Gobiodon* sp.3) and conspicuously coloured (*G*. *histrio*) *Gobiodon* species in the Red Sea release skin secretions when stressed. These secretions impact the locomotory abilities of both potential space competitors and predators, suggesting a high toxicity and confirming earlier studies (Hashimoto *et al*. 1974; Lassig 1981; Munday *et al*. 2003; Schubert *et al*. 2003). *Gobiodon unicolor*, the cryptically coloured representative used in bioassay studies by Schubert *et al*. (2003), is identical to *G*. sp.3 (based on morphological and molecular data; J. Herler, unpublished data), and both species have been recently united under the name Gobiodon fuscoruber (Herler, Bogorodsky & Suzuki 2013). Thus, our bioassay experiments are fully comparable with those of Schubert *et al*. (2003), who found interspecific differences in toxicity related to coloration. In contrast, Hashimoto *et al*. (1974) found no major difference between *Gobiodon* species. In the present study, the cryptically coloured *G*. sp.3 was somewhat less toxic than *G. histrio*. Different toxicity levels of the same species may reflect regional differences. The toxicity of gobies may also be enhanced by the ingestion of toxic algae (Dixson & Hay 2012). Additionally, the rapid drop in toxicity over time may play a role: our experiments revealed differences in species-specific toxicity only until 10 min after mucus production. Toxins remained very potent for up to 30 min, and only after 60 min did the first bioassay specimens no longer lose equilibrium. This may reflect enzymatic degradation (Hashimoto *et al*. 1974) or bacterial detoxification (Thomson 1964). The toxic effect of gobies seems to be relatively similar on different bioassay species. The damselfish *C. viridis* in the present study lost its equilibrium about 60–120 s after exposure to fresh toxins. Very similar values were obtained for the apogonids *Apogon fragilis* Smith (mean of 100 s), *Ostorhinchus nigrofasciatus* (Lachner) (66–143 s) and *Nectamia similis* Fraser (65–162 s) (Schubert *et al*. 2003; Dixson & Hay 2012). These relatively constant and high levels of toxicity suggest that predation pressure is generally high for coral gobies. Nonetheless, *G*. sp.3 may be less exposed due to its lower movement rate between corals and its structurally more complex host coral (J. Herler, personal observations; Dirnwoeber & Herler 2012), reducing the investment in skin toxins (Williams *et al*. 2003). Accordingly, this study focused mainly on how a *Gobiodon* predator would probably deal with a coral goby under natural conditions. Post-capture behaviour of predators contrasted sharply with pre-capture behaviour. Prior to capture, *E. fasciatus* was equally interested in all three prey species and did not show clear preferences, suggesting that gobies are potential prey in their natural environment. Choices for consumption became explicit only during post-capture behaviour. *Chromis viridis* was not only consumed most often, but was also usually consumed immediately without being expelled after capture. As in other toxic or unpalatable species (Bakus 1981; Wiklund & Jaervi 1982), both gobies experienced repeated expulsion after being *‘*sampled*’*. They always survived quick regurgitation, as did other toxic species elsewhere (Bakus 1981; Wiklund & Jaervi 1982).This provides evidence for much higher survival rates and related fitness of crinotoxic fishes. Despite preceding rejections, 10 of 30 coral gobies were finally consumed after a minimal cumulative manipulation time of more than 80 s. The cumulative manipulation time of rejected gobies was much less than that of gobies that were finally eaten. This indicates that a minimum manipulation time of approximately 1.5 min is required before predators are willing to consume gobies. This result supports Lassig (1981), who also noted that gobies were rarely swallowed without prior manipulation. We observed that predators that did not consume gobies made this decision after a few manipulations/very short manipulation time. This implies quick learning about goby unpalatability/toxicity.

Rapid expulsion (a few seconds) after first capture infers either gobiid unpalatability or quickly determined toxicity by the predator, or both, in agreement with Hashimoto *et al*. (1974). Prey regurgitated after brief manipulation has a very high chance of survival. Although repeated prey capture may be difficult for a predator in the field, a close association with the substrate (coral host) no doubt provides long-term protection and enhances survival rates (Feary *et al*. 2007; Wall & Herler 2009). Despite goby unpalatability, one would expect the ingestion of *C. viridis* to be more difficult, as it has full body squamation and robust fin spines. Nevertheless, its manipulation time before consumption was much shorter than for gobies. The repeated regurgitation of gobies and the prey preferences of predators suggest that chemical deterrence, particularly if combined with toxicity, is much more efficient than mechanical/physical deterrence. This is of particular interest because some coral fishes apparently increase their toxicity through their diet (Dixson & Hay 2012). In our experiments, the toxins had no visible effect on *E. fasciatus* after digestion of gobies [even after observations overnight, agreeing with Schubert *et al*. (2003) and Lassig (1981)]. Nonetheless, exposure to goby skin secretions caused a loss of equilibrium. This indicates that the toxins lose their haemolytic property upon digestion and primarily target the blood circulation via the gills of predators, resulting in haemolysis (Primor & Zlotkin 1975). Gobies were very rarely swallowed immediately at their first capture, but were frequently and repeatedly expelled. Our interpretation is that the toxin is most effective shortly after capture and before ingestion, preventing the prey from being swallowed.

Predator preferences may be influenced not only by distance or olfactory stimuli but also by prey coloration. However, our experiments revealed no clear preference for either *Gobiodon* species and thereby for any type of coloration. This suggests no avoidance mechanism associated with signal colour. Furthermore, predators apparently preferred prey with high condition factors (as revealed by Cox-regression). Due to the negative correlation between condition factor and body length in gobies, we assume that small gobies are at a particularly high risk of being eaten: the smaller fish are easier to handle and less toxic, based on the significant positive correlation between length and toxicity (at least in *G. histrio*). Another factor possibly affecting a prey choice during experiments is naivety towards the prey species; the results may be biased if a predator had previous encounter experiences with one or more of the experimentally used species. Nevertheless, we show that, although the groupers preferred other prey, they did not show explicit pre-capture avoidance to gobies and a few ultimately consumed them. Therefore these predators are stimulated by gobies and are eager to consume them.

In summary, the present study supports and extends earlier studies showing that skin toxins in *Gobiodon* have a range of biological functions, including parasite and predator deterrence (Lassig 1981; Munday *et al*. 2003; Schubert *et al*. 2003). Recently, Dirnwoeber & Herler (2012) showed that these toxins are even involved in coral predator deterrence. It is unusual for the chemical composition of an ichthyocrinotoxin to have such a wide ecological functionality. This calls for toxicological research into these toxins to determine their nature (either grammistin or pahutoxin, as suggested by Hashimoto *et al*. 1974). More field research and experiments are also necessary to evaluate additional roles the toxins may play within the environment of *Gobiodon,* including the influence of life stage and migration frequency on toxicity levels and the role of toxicity as a potential deterrent of space competitors.
